# Supplementary material for: Unveiling the Role of Protein Kinase C θ in Porcine Epidemic Diarrhea Virus Replication: Insights from Genome-Wide CRISPR/Cas9 Library Screening
Source: Int J Mol Sci. 2024 Mar 7;25(6):3096. doi: 10.3390/ijms25063096 (PMC10969977; doi:10.3390/ijms25063096)
Supplement: Supplementary file 1 [file ijms-25-03096-s001.zip › ijms-2872561-supplementary.pdf]

Supplementary Material

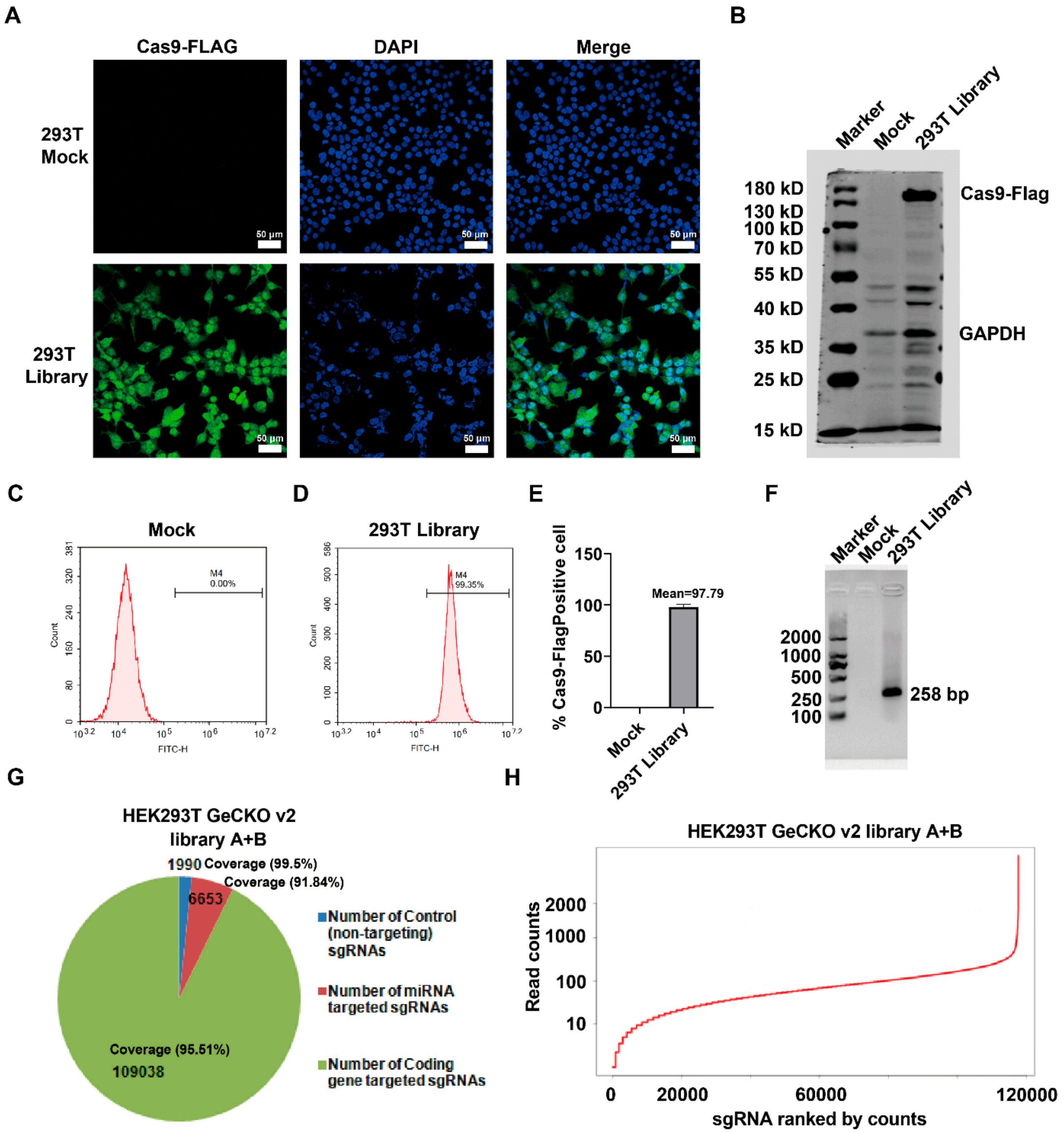

Supplementary Figure S1. Evaluation of genome-scale CRISPR screening of host factors associated with PEDV infection. (A) Confocal microscopic images of HEK293T cells and Cas9-Flag-transfected HEK293T cells. Scale bars, 50 µm. Data are presented as the mean ± SD of at least three independent experiments. **(B)** Representative Western blot images showing stable expression of Cas9-Flag protein in HEK293T cells compared to control HEK293T cells. **(C)** FACS analysis of HEK293T

cells without Cas9-Flag transfection. **(D)** FACS analysis of HEK293T cells transfected with Cas9-Flag. **(E)** The proportion of HEK293T cells and Cas9-Flag-transfected HEK293T cells. **(F)** PCR amplification of human GECKO library. **(G)** Pie chart illustrating the amplicon sequencing results from the GECKO library of HEK293T cells. Blue, red, and green colors represent numbers of non-target sgRNAs, miRNA targeted sgRNAs, and coding gene targeted sgRNAs, respectively. **(H)** Line chart depicting the homogeneity of sgRNAs in the GECKO library of HEK293T cells.
